# The Effectiveness and Optimal Dose of Resistance Training in Patients With Subacute and Persistent Low Back-Related Leg Pain: A Systematic Review

**DOI:** 10.7759/cureus.57278

**Published:** 2024-03-30

**Authors:** Valerio Barbari, Maria M Carbone, Lorenzo Storari, Marco Testa, Filippo Maselli

**Affiliations:** 1 Department of Human Neurosciences, Sapienza University of Rome, Rome, ITA; 2 Department of Neuroscience, Rehabilitation, Ophthalmology, Genetics, Maternal and Child Health, University of Genova, Savona, ITA

**Keywords:** systematic review, quality of life, disability, pain, resistance training, lblp

## Abstract

A subgroup of patients with low back pain (LBP) suffers from low back-related leg pain (LBLP), which can be classified as radicular pain, or somatic referred pain without nerve root involvement. LBLP is considered an obstacle to recovery and a strong negative prognostic factor for medium- and long-term disability. In this review, we aimed to investigate the effectiveness and optimal dose of resistance training (RT) in patients with subacute or persistent LBLP to provide clinical recommendations for practice. This systematic review was conducted by adhering to the Preferred Reporting Items for Systematic Reviews and Meta-Analyses (PRISMA) guidelines and the recommendations of the Cochrane Collaboration.

We conducted a literature search on PubMed, PEDro, Cochrane Library, Scopus, and Web of Science databases. Only randomized controlled trials (RCTs) involving patients ≥18 years of age were included. The risk of bias in the included studies was assessed using "the Cochrane Collaboration's tool for assessing risk of bias" (RoB) and the inter-rater agreement for full-text selection was evaluated using Cohen's Kappa (K). The search elicited a total of 4.537 records, and two RCTs involving a total of 196 participants were identified through a selection process based on title, abstract, and full-text assessment. Both studies had a low to moderate risk of bias. The inter-examiner concordance index for the selection of full text was excellent (K=1). RT seems to be an effective and safe intervention for patients with LBLP, but its long-term effectiveness, superiority over other types of exercise-based therapies, and optimal dosage still constitute a gray area in the literature.

## Introduction and background

Low back pain (LBP) is an extremely common health problem: it has been considered the leading cause of years lived with disability since 1990 and a growing global public health burden [1,2]. The Global Burden of Disease 2010 study confirmed that LBP had a global prevalence of 9.4%, with men more affected (10.1%) compared to women (8.7%). The same study also showed that its prevalence was higher in Western Europe (15.0%), followed by North Africa/Middle East (14.8%/6.5%), and Central and Latin America (6.6%). In Brazil, a systematic review (SR) showed that the seven-day prevalence rate was 4.2-31.4% and, in the last year, it was 13.1-66.8%. It also revealed that the prevalence rate in adults in the last year was greater than 50%, while it ranged from 13.1% to 19.5% in adolescents. The data of one-time prevalence was up to 84% and chronic pain frequency was about 23% [3-5].

A subgroup of patients with LBP, accounting for about 60% of cases, also report related lower extremity pain, known as low back-related leg pain (LBLP) [6], which is associated with a worse prognosis, high care-seeking and healthcare-related costs, and higher level of disability [7]. To guide the diagnosis and prognosis of LBLP, several clinical classifications have already been proposed in the literature, such as patho-mechanism-based approach [8], pain mechanism dominance, results of neurological examinations, or systems based on pain-generators on nerve-related versus somatic-referred symptoms [9-17]. However, Stynes et al. concluded that the validity of classification systems scores is still poor, particularly in terms of content and construct validity [18]. Irrespective of all the classification systems, the most common diagnostic framework of LBLP is the one based on three main clinical conditions: (1) referred leg pain (pain arising from somatic structures, such as ligaments, joints, tendons, discs, and/or muscles; (2) nerve-related leg pain (radiculopathy with or without radicular pain with pain radiating down to the leg associated with symptoms such as paresthesia, burning, tingling with or without muscle weakness, impaired osteo-tendon reflexes, and/or sensory deficit depending on the results of the neurological examination); and (3) mixed conditions in which referred pain and nerve-related leg pain may coexist - according to preliminary evidence [19-21].

Nevertheless, it should be considered that LBLP is associated with greater disability, poor prognosis, worse quality of life, and higher health-related costs than LBP alone regardless of the patho-anatomical process [22]. Therefore, in the domain of LBLP, clinicians and researchers are still dealing with the uncertainty of both the diagnosis and - according to the alarming prognostic pattern of LBLP elicited from prospective studies - the management of this complex clinical condition. Concerning the treatment of the wider population of LBP patients, current guidelines recommend that the first line of management may be mediated by conservative therapies and mainly by therapeutic exercises (TE), manual therapy (MT) associated with TE, and psychological treatments [23-26].

However, even though several types of exercise have already been proposed for chronic LBP (CLBP) patients with or without LBLP - such as motor control [27], core stability exercises [28], stretching [29], and aerobic exercise [30] - the most effective type of exercise remains unclear [31]. Furthermore, for the specific condition of LBLP, previous SRs investigating the effectiveness of exercise-based interventions mainly focused on patients with sciatica (radicular pain or radiculopathy) [32,33], but nothing is known regarding the clinical condition of LBP with somatic referred leg pain. The latter aspect may be due to the assumption that LBP with LBLP with somatic-referred origin has globally been considered to be the same clinical and pathological condition of LBP without leg pain (non-specific LBP) - according to all previous triages proposed in literature which classify three clinical conditions: (1) non-specific LBP; (2) specific LBP (radicular syndromes such as radicular pain, radiculopathy, or spinal stenosis); and (3) medical conditions [34-40]. However, since LBLP has a higher disabling and prognostic impact than LBP alone, previous considerations still represent uncovered areas of research. Also, to the best of our knowledge, no clinical practice guidelines for the specific management of both somatic and radicular LBLP patients currently exist.

Among the several forms of exercise proposed in the management of LBP with or without leg pain, resistance training (RT) (training or exercise against resistance such as weights, rubber bands, water, or immovable objects that results in progressive overload of musculoskeletal tissues [41]) has been gaining significant attention from a research perspective. In particular, the beneficial effects of RT have already been demonstrated in different populations with musculoskeletal or medical conditions, such as the elderly [42,43], individuals with type 2 diabetes [44], osteoporosis [45], osteoarthritis [46,47], chronic neck pain [48], fibromyalgia [49], and patients with CLBP [50] - indicating its benefits and use in clinical practice. However, to the best of our knowledge, no SRs have investigated the effectiveness of RT in the specific population of patients with LBLP - regardless of the pathoanatomical pain generators. Therefore, the objective of this SR was to investigate the effectiveness of RT on clinically relevant outcomes such as pain, disability, and quality of life in patients with subacute and persistent LBLP.

## Review

2. Methods

This systematic review was conducted in line with the Preferred Reporting Items for Systematic Reviews and Meta-Analyses (PRISMA) and Cochrane Collaboration guidelines (Cochrane Handbook 5.1.0). The protocol has been registered with Prospero (CRD42022355998) [51,52].

2.1. Eligibility Criteria

2.1.1. Study design: Only randomized controlled trials (RCTs) published in English were eligible. No restrictions related to the publication date were applied.

2.1.2. Participants: Participants with subacute (pain between 4 and 12 weeks) or chronic (pain beyond 12 weeks) LBLP regardless of its nature (referred, nerve-related, or mixed pain) were included. Temporal classification of subacute and chronic pain has been chosen in line with previous taxonomies [25,53]. All studies involving participants younger than 18 years of age and/or had pathology beyond the physical therapy scope of practice (for example, cauda equina syndrome, tumors, spinal fractures, or any other medically relevant pathology underlying symptoms in the spine and lower extremity) were excluded. Also, participants with acute LBLP were excluded because we assumed that RT may not represent the first line of interventions in the rehabilitative context for those patients, mainly due to the potential irritability and severity of symptoms.

2.1.3. Interventions: Studies where RT was used as a single intervention strategy or combined with other interventions were included. In detail, all types of RT modalities were deemed eligible - such as weights (dumbbells, barbells), rubber bands, immovable objects, bodyweight exercises, or any other modalities involving the execution of exercises against resistance.

2.1.4. Comparisons: Usual care, usual physiotherapy, MT, no intervention, placebo interventions, waiting lists, and other forms of exercise different from RT or any other treatment modalities were all eligible for inclusion.

2.1.5. Outcome and outcome measures: To be eligible, RCTs had to assess at least one of the following three outcomes: (1) pain, (2) disability, or (3) quality of life - as assessed by objective measures, patient-reported questionnaires, or other modalities.

2.2. Search Methods for the Inclusion of Studies

2.2.1. Electronic searches: An electronic search was performed between June and December 2022 on PubMed, PEDro, The Cochrane Library, Scopus, and Web of Science. The search strategies were created depending on the specific settings of each database. The search strings were developed according to the PI(C)(O) model of clinical questions (participants, interventions). To make the search strategies sensitive, we did not insert keywords for comparisons and outcomes. Where possible MeSH (Medical Subject Headings) terms were used and combined with Boolean operators (AND, OR, NOT). Additionally, we conducted a manual search of all bibliographies of the studies assessed for the subsequent full-text selection and references obtained from 11 systematic reviews focused on LBP and LBLP.

2.3. Study Selection and Data Extraction

After the removal of duplicates, articles were initially screened through titles and abstracts. Then, full-text articles considered potentially eligible for inclusion were analyzed and subsequently selected according to the inclusion criteria. The selected full-texts were independently screened by the main two reviewers (VB, MMC), and included in the review. In case of disagreements, a third author (LS) not involved in the full-text selection process was consulted. Finally, articles deemed eligible after reading the full texts were evaluated for potential risk of bias. Data were extracted from each article by using a standard data extraction system in line with the P.I.C.O. (P: Participants; I: Intervention; C: Control; O: Outcome)) model of the clinical research question, the research protocol, the PRISMA guidelines [51], and the Cochrane Handbook (https://training.cochrane.org/handbook/current/chapter-08) recommendations. Data extraction, therefore, was organized according to the following parameters:

· General information: author, publication date, and study design;

· Participants: duration of pain, sample size, age and sex of participants, and diagnostic criteria of patients;

· Interventions/controls: content, procedures, and frequency and duration of interventions;

· Outcome: type of outcome and outcome measures;

· Follow-ups.

2.4. Inter-rater Agreement

Cohen’s Kappa (K) was used to quantify the inter-rater agreement among authors for full-text selection. Cohen’s K was interpreted according to Altman’s definition: k<2: poor, 0.2<k<0.4: fair, 0.41<k<0.60: moderate, 0.61<k<0.80: good, and 0.81<k<1.00 excellent [54].

2.5. Risk of Bias

The Cochrane Collaboration tool [55] was independently used by the two main authors (VB, MMC) to assess the risk of bias (RoB) of the included studies. Then, the ratings of the two reviewers were compared, and discrepancies were resolved with a third reviewer (FM) not involved in the RoB assessment process.

3. Results

**Figure 1:**
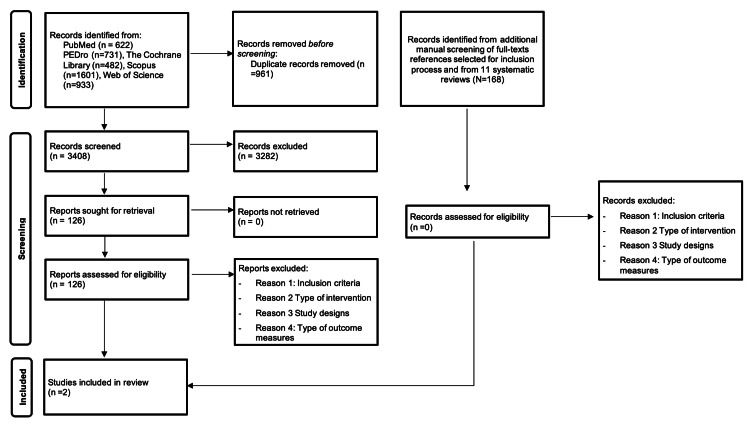
Flow chart depicting the study selection process

Electronic database searches yielded 4369 results. After removing 961 duplicates, 3282 records were excluded based on the assessment of titles and abstracts, leaving 126 studies eligible for full-text evaluation. Subsequently, 124 full-text articles were removed because they did not meet the inclusion criteria. Through manual search, we identified an additional 168 potentially relevant references. On screening, none of them met the eligibility criteria. The complete search process is shown in Figure 1.

3.1. Study Characteristics

A total of two RCTs were included [56,57]. The study settings, countries, recruitment facilities, diagnostic criteria, sex, age, duration of LBLP, and number of participants are shown in Table 1.

**Table 1:** Characteristics of the included studies RCT: randomized controlled trial; CLBP: chronic low back pain; M: male; F: female; PILE: progressive isoinertial lifting evaluation; ODI: Oswestry Disability Index; VAS: visual analogue scale; NRDP: non-reducible discogenic pain; I: intervention group; C: control group; IFR: individual functional restoration; NRS: numeric rating scale; Δg: between-group difference values

General information (author, year, study design, country)	Population (characteristics, number, age, gender, recruitment)	Interventions (number of participants, content, frequency, duration)	Comparisons (number of participants, content, frequency, duration, and professional in charge)	Outcomes and outcome measures	Results and mean values for outcome measures for each follow-up [Absolute values (Limke et al.)]; means ± SD (Chan et al.) for experimental groups (EX) and control groups and between-group difference values (Δg)
Limke et al., 2008 [56] RCT USA	CLBP > 3 months with or without leg pain N = 100 Age (I) = 47 years Age (C)= 46 years M= 36; F= 64 Patients referred to hospital for a structured group physical therapy program	N = 51 1 set of resistive exercises: group therapy sessions (maximum 12 patients) twice a week for an average of 6 weeks, lasting 1.5 hours No hands-on treatments 15 minutes of stretching 2/3 times a day	N = 49 2 sets of resistive exercises: same training protocol as the experimental group by performing, however, two sets of each exercise)	Primary outcomes strength in back extension progressive isoinertial lifting (PILE) Secondary outcomes disability (ODI) pain (VAS) Follow-up Baseline 6 weeks	ODI EX: 38 → 21 C: 38 → 22 Back pain (VAS) EX: 6 → 4 C: 5 → 3 Leg pain (VAS) EX: 3 → 3 C: 4 → 3
Chan et al., 2017 [57] RCT Australia	CLBP with indicative NRDP characteristics from a minimum of 6 weeks to a maximum of 6 months N = 96 Age (I)= 42.3 years Age (C)= 41.8 years M=46; F= 50 Patients were recruited through advertisements, public notices, or referred by physicians and health professionals	N = 50 Individualized Functional Restoration (IFR): 10 30-minute treatment sessions for 10 weeks Education, training program (resistance training), and cognitive-behavioral treatments	N = 46 Advice group: 2 counseling sessions with a physical therapist lasting 30 minutes. The third session scheduled after 5 weeks	Primary outcomes Back pain (NRS) Leg pain (NRS) Activity limitation (ODI) Secondary outcomes General Health (EuroQol-5D thermometer) Sciatica Frequency and Bothersomeness Score Psychosocial score (Örebro) Follow-up Baseline 5 weeks 10 weeks 26 weeks 52 weeks	Back pain intensity (NRS) EX: 5.7 ± 1.8 → 3.7 ± 2.2 → 3.0 ± 2.3 → 3.1 ± 2.4 → 2.8 ± 2.7. C: 5.7 ± 1.8 → 4.4 ± 2.5 → 4.3 ± 2.6 → 4.0 ± 2.5 → 3.3 ± 2.3 Δg 5 weeks: 0.6 (−0.2 to 1.5) Δg 10: 1.1 (0.1 to 2.1) Δg 26: 1.0 (−0.1 to 2.0) Δg 52: 0.4 (−0.7 to 1.5) Leg pain intensity (NRS) EX: 4.7± 2.9 à; .3.0 ± 2.7 à; 2.1 ± 2.5 à; 2.3 ± 2.6 à; 1.9 ± 2.6. C: 4.7 ± 2.5 à; 4.0 ± 2.8 à; 3.7 ± 2.8 à; 3.3 ± 3.0 à; 2.3 ± 2.5. Δg 5:0.9 (−0.2 to 2.0) Δg 10:1.5 (0.4 to 2.6) Δg 26:0.8 (−0.3 to 1.9) Δg 52:0.2 (−0.9 to 1.3) ODI EX: 30.3 ± 11.0 à 25.0 ± 12.7 à 17.6 ± 12.6 à 16.5 ± 12.9 à 16.2 ± 14.6. C: 30.3 (11.8) à 26.8 (17.6) à 24.4 ± 15.8 à 22.8 ± 18.1 à17.3 ± 14.2. Δg 5:1.8 (−3.2 to 6.8) Δg 10:6.3 (1.3 to 11.4) Δg 26:6.6 (1.4 to 11.8) Δg 52:1.1 (−4.0 to 6.2)

3.1.1. Study design: The included studies were RCTs published in English in 2008 [56] and 2017 [57]. One study was a parallel-group RCT [56], while the other was a multicenter parallel-group RCT [57].

3.1.2. Drop-outs and loss to follow-up: The total number of dropouts was 20. In the study by Limke et al., 16 subjects dropped out before completing the treatment program (six due to non-compliance with the therapy program, two participants chose to schedule therapy once a week and were excluded from the study, one was pregnant, one was sent for a surgical consult, one was excluded due to personal reasons, one due to scheduling difficulties, three due to unrelated medical problems, and one due to a spine-related medical problem); of note, the drop-out rate was 18% for the experimental group and 20% for the control group [56]. In Chan's study, four subjects dropped out before completing the treatment program, with a dropout rate of 2% for the experimental group and 6.5% for the control group. In the same study, five subjects were lost to follow-up (two in the experimental group and three in the control group). In this study, no reasons for dropouts or loss to follow-up were provided [57].

3.1.3. Follow-ups: The study by Limke et al. involved a post-intervention follow-up (six weeks) [56], while the study by Chan et al. verified the results at 5, 10, 26, and 52 weeks after randomization [57].

3.1.4. Adverse effects: No serious adverse events were reported in either study.

3.1.5. Type of participants: Participants were all adults aged 18-65 years with nonspecific CLBP with or without leg pain. The total number of patients recruited and then randomized was 212, and 192 attended all follow-ups (90%). In the study by Limke et al. (100 participants completing the study), 36 participants were male and 64 were female. The average age of the experimental group was 47 years, while that of the control group was 46 years. In addition, at the baseline, 93% of the 51 participants in the experimental group and 87% of 49 participants in the control group also reported leg pain [56]. In the study by Chan et al., however, among the 50 patients randomly placed in the experimental group, 24 were male and 26 were female and the mean age was 42.3 years; among the 46 patients in the control group, 22 were male and 24 were female and the mean age was 41.8 years. Also, 80 participants had back pain with associated leg pain at baseline and subsequent follow-ups [57]. All characteristics of participants are given in Table 1.

3.1.6. Type of interventions and type of control group: The interventions used in the studies and their respective control groups have been detailed in Table 1.

3.1.7. Type of outcome and outcome measures: Of the three main outcomes considered in this systematic review, pain and disability were considered secondary outcomes in the study by Limke et al. [56], whereas in the study by Chan et al., pain and disability were considered primary outcomes [57]. Outcomes and relative measures are reported in Table 1.

3.2. Risk of Bias

Methods to ensure effective randomization were appropriate in both studies. In the study by Limke et al., the lack of blinding of the staff offering the interventions was judged to be at high risk of bias [56]; similarly, the inability to blind patients and healthcare staff makes the study by Chan et al. at high risk of bias [57]. Only in Chan et al.'s study, the questionnaire data were assessed by a researcher blinded to the allocation of participants and that the intention-to-treat analyses were carried out adhering to established protocols [57]. Both RCTs adhered to their own study protocol and methods, reporting results against all previously stated outcomes - then, the risk of a reporting bias was low. The "other" section was rated as low risk of bias for both studies. The individual RoB item ratings for each study are shown in Table 2.

**Table 2:** Risk of bias in the included studies

	Random sequence generation (selection bias)	Allocation concealment (selection bias)	Blinding of participants and personnel (performance bias)	Blinding of outcome assessment (detection bias)	Incomplete outcome data (attrition bias)	Selective reporting (reporting bias)	Other bias
Limke et al., 2008 [56]	Low risk	Low risk	High risk	High risk	Uunclear Risk	Low risk	Low risk
Chan et al., 2017 [57]	Low risk	Low risk	High risk	Low risk	Low risk	Low risk	Low risk

3.3. Agreement

The inter-examiner agreement between the authors was excellent (Cohen's K=1) for full-text selection. The related data are presented in detail in Table 3.

**Table 3:** Inter-rater agreement between authors for full-text selection

Agreement for full-text selection		Author 1 (MMC)		Total
		Positive evaluation	Negative evaluation	
Author 2 (VB)	Positive evaluation	2	0	2
	Negative evaluation	0	290	290
Total		2	290	292

3.4. Effectiveness of Interventions

Although there was a trend towards improvements for all outcomes in the study by Limke et al., there were no significant differences at six weeks for disability, leg pain, and pain. Also, all improvement values were almost similar between both groups over time [56]. However, in the study by Chan et al., both groups achieved significant mean improvements in pain and activity limitation. Comparisons on primary outcomes showed greater improvements in all primary measures favoring the experimental group with a statistically significant between-group difference at 10 weeks: back pain intensity (NRS 0-10; p=0.026), leg pain intensity (NRS 0-10; p=0.009), activity limitation (Oswestry Disability Scale, p=0.014); and at 26 weeks for activity limitation (Oswestry Disability Scale, p=0.013). There were significantly greater between-group improvements favouring the experimental group on all continuous secondary outcomes excluding EuroQol-5D (health score) at 10 weeks. The experimental group also showed greater improvements on the Sciatica Bothersomeness Score at five (p=0.042) and 26 weeks (p=0.041). Ordinal secondary outcomes favored the experimental group for the global rating of change and satisfaction scores at five (p=<0.001), 10 (p=<0.001), and 26 weeks (p=0.009). However, no differences were found between the groups for work and EuroQol-5D (utility score) [57]. Details of the effectiveness of interventions are presented in Table 4.

**Table 4:** Effectiveness of interventions ←: effect in favour of experimental intervention; →: effect in favour of control group; ODI: Oswestry Disability Index; VAS: visual analog scale; IFR: individual functional restoration; NRS: numeric rating scale; SBS: Sciatica Frequency and Bothersomeness Scale

Study	Experimental intervention	←	No between-group difference	→	Control group	Outcomes
Limke et al., 2008 [56]	1 set of resistive exercises		ODI p= 0.87 VAS (Back pain) p= 0.44 VAS (Leg pain) p= 0.49		2 sets of resistive exercises	VAS (Back pain) VAS (Leg pain) ODI
Chan et al., 2017 [57]	Individualized functional restoration (IFR)	NRS (Back pain) p = 0.026** NRS (Leg pain) p= 0.009** ODI p= 0.014** p= 0.013*** SBS p=0.042* p=0.002** p=0.041***	NRS (Back pain) p= 0.16* p= 0.08*** p= 0.4**** NRS (Leg pain) p= 0.11* p= 0.17*** p= 0.75**** ODI p= 0.48* p=0.67**** SBS p= 0.65**** EuroQol-5D p= 0.15* p=0.15** p=0.13*** p=1.00****		Advice	NRS (Back pain) NRS (Leg pain) ODI SBS EuroQol-5D

4. Discussion

To the best of our knowledge, this is the first SR investigating the effectiveness of RT in patients with subacute or persistent LBLP. However, this topic is still a gray area in the literature, and this aspect was concretely reflected in the low number of RCTs included in this SR. Overall, both RCTs demonstrated the beneficial effects of RT on all outcomes, although the intervention modalities differed considerably between the two studies. In detail, one was based solely on therapeutic exercise-aerobic training, stretching, and RT [56], while the other one was based on a multimodal approach characterized by education, exercise program (RT), and cognitive-behavioral treatments [57]. Overall, both studies were rated as low risk of bias. In detail, the study by Limke et al. showed that there was no difference between performing one or two sets of exercise, and improvements in both groups were almost similar. Therefore, the choice related to the dosage of RT in patients with LBLP still relies on clinical expertise. Also, it must be considered that primary outcomes were back strength and progressive isoinertial lifting evaluation (PILE) at discharge, while pain and disability were set as secondary outcomes. Then, the sample size was calculated based on the surrogate and physical outcome of back strength, and it is not clear if the study should be considered without any bias in these terms.

The study by Chan et al. demonstrated the superiority of individualized functional restoration (IFR) for all outcome measures compared to advice alone. However, it must be kept in mind that the choice of comparator is a major and critical aspect of the design of RCTs. Indeed, the study of Chan et al. shows that IFR is superior to advice only, but nothing is known about the effectiveness of IFR compared to other forms of active treatments. Although the findings of this SR seem promising, and since it should be emphasized that RT-based treatment is certainly effective for CLBP patients with and without leg pain based also on our results, the best modalities of exercise-based interventions are still unknown [31]. Therefore, starting from the preliminary benefits that emerged from this SR, further high-methodological quality studies should be conducted to investigate the effectiveness of RT in this population, to compare it with other forms of exercise, and to detect the best dosage of exercise training.

4.1. Applicability

Although all included participants could be identified as CLBP patients with or without leg pain, the inclusion criteria adopted by the included studies were extremely specific concerning both age (18-65 years), duration of pain and clinical presentation, and certain thresholds of scores regarding disability (e.g., ODI >20%). Although such specificity of inclusion criteria may reflect for researchers the main standard modalities of recruitment in primary studies, it may circumscribe the generalizability of results to a limited number of patients only. However, the settings in which the studies took place were hospital settings and physical therapy clinics - reflecting the real health facilities where clinicians may encounter patients with subacute and persistent LBLP.

Regarding intervention modalities, there was no homogeneity among the programs both in frequency and duration and in terms of the type of RT-based exercises proposed. Since one of the main critical problems in CLBP rehabilitation is the research into optimal doses of exercise, the heterogeneity of intervention programs may significantly limit the transferability of results in clinical practice. It is important to note that in one study [56] follow-up was established only in the short term (six weeks) and this aspect lends significant uncertainty to the results obtained, invalidating its immediate transferability to a clinical setting. The absence of adverse events related to the interventions and the administration of RT exclusively by physical therapists are surely two major strengths for the transferability of results. In line with these considerations, it may be hypothesized that the findings of this SR are not affected by significant limits for applications in a real-world outpatient setting.

4.2. Consistency

To the best of our knowledge, this is the first SR investigating the effectiveness of RT in patients with subacute and persistent LBLP. Consequently, the real consistency is markedly more limited than other SRs focused on the effectiveness of exercise in patients with CLBP with or without leg pain and it is still inexistent. For these reasons, the following discussion related to the consistency of our results is essentially limited to the wider and more general spectrum of research focused on exercise and patients with LBLP. Considering patients with LBLP of radicular origin, our results are in line with all other papers in the scientific scenario of the effectiveness of therapeutic exercise in radiculopathy and/or radicular pain [32,58,59]. Patients with LBLP, however, should also be considered as part of the subgroup of nonspecific LBLP according to the widely accepted triages; our results are surely in line with all SRs supporting the effectiveness of exercise-based treatments among which RT is also included [28-30,46,60-62]. Therefore, in line with previous considerations, it may be asserted that our results are generally consistent with previous and current research.

4.3. Strengths and Limitations

This review has a few limitations. In detail, the fact that we considered publications in Italian and English languages only, the absence of research on individual peer-review journals, and the absence of meta-analysis due to the heterogeneity of intervention modalities, control groups, and outcome measures must be considered limitations. However, the high sensitivity of search strategies to include as many studies as possible (five electronic databases and large manual bibliographic screening), the excellent K score, and the fact that this SR is the first work on this topic should be considered the major strengths of our work. Furthermore, this is the first SR specifically focusing on LBLP. Therefore, various implications for future research may emerge based on our results.

4.4. Implication for Further Research

Based on the findings of our SR, we recommend that future primary studies:

- Establish long-term follow-up;

- Specifically include patients with LBLP;

- Distinguish and subgroup patients with different leg pain origins (radicular, non-radicular or referred, and mixed);

- Standardize intervention procedures.

4.5. Implications for Clinical Practice

RT seems to be an effective and safe intervention, but there is a paucity of RCTs supporting its transferability in clinical practice. While exercise-based interventions remain effective and recommended methods in the management of patients with musculoskeletal lower back disorders, for RT, no clinical recommendations can be made regarding the optimal dosage (intensity, frequency, duration, time, sets, and repetitions).

## Conclusions

RT is currently considered an effective intervention in patients with musculoskeletal diseases. While the scientific premise for this approach in patients with CLBP seems to be clear, findings of the effectiveness of RT in the specific subgroup of patients with LBLP are still extremely narrow and, at best, promising due to the paucity of studies. However, based on the two RCTs included in this SR, RT seems to be effective regarding outcomes related to improved pain, disability, and quality of life in patients with LBLP irrespective of leg pain origin. To elaborate, RT showed an overall and positive trend towards improvements in any outcome related to clinically relevant measures irrespective of administration modalities, and, as per our results, there may be no differences in terms of exercise volume (one versus two sets) to achieve such improvements. The latter aspect may be important in clinical scenarios and could enable clinicians to modulate intensity, volume (sets, repetitions), or any other parameters of RT based on the clinical characteristics of patients (age, deconditioning).

Therefore, the absence of a statistically significant difference between the two proposals (one versus two sets) may be perceived as a positive impact on clinical practice. Furthermore, RT is, at worst, statistically similar to educational advice-based interventions. Although the comparison between exercises and education may be not informative in a conclusive manner (since both interventions are strongly recommended by clinical practice guidelines for the management of patients with LBP with or without leg pain), such results directly testify that RT, and exercise-based interventions in general, are predominantly required in LBP rehabilitation and that education alone may not be sufficient for main and clinically relevant outcome measures. However, further larger high-quality studies are required to explore the effectiveness of RT in patients with LBLP and to provide consistent recommendations regarding intervention modalities and the optimal dose to implement RT itself in clinical practice based on specific clinical profiles (radicular pain or radiculopathy, non-specific LBP with somatic referred leg pain, or mixed conditions).

## Appendices

**Table 5:** PRISMA 2020 checklist* *[63] For more information, visit http://www.prisma-statement.org/

Section and topic	Item #	Checklist item	Location where the item is reported
Title			
Title	1	Identify the report as a systematic review	Lines 2-3
Abstract			
Abstract	2	See the PRISMA 2020 for the Abstract checklist	Lines 10-27
Introduction			
Rationale	3	Describe the rationale for the review in the context of existing knowledge	Lines 30-95
Objectives	4	Provide an explicit statement of the objective(s) or question(s) the review addresses	Lines 96-98
Methods			
Eligibility criteria	5	Specify the inclusion and exclusion criteria for the review and how studies were grouped for the syntheses	Lines 103-131 Lines 143-160
Information sources	6	Specify all databases, registers, websites, organizations, reference lists, and other sources searched or consulted to identify studies. Specify the date when each source was last searched or consulted	Lines 132-142
Search strategy	7	Present the full search strategies for all databases, registers, and websites, including any filters and limits used	Lines 132-142
Selection process	8	Specify the methods used to decide whether a study met the inclusion criteria of the review, including how many reviewers screened each record and each report retrieved, whether they worked independently, and if applicable, details of automation tools used in the process	Lines 143-160
Data collection process	9	Specify the methods used to collect data from reports, including how many reviewers collected data from each report, whether they worked independently, any processes for obtaining or confirming data from study investigators, and if applicable, details of automation tools used in the process	Lines 145-160
Data items	10a	List and define all outcomes for which data were sought. Specify whether all results that were compatible with each outcome domain in each study were sought (e.g. for all measures, time points, analyses), and if not, the methods used to decide which results to collect	Lines 128-131
	10b	List and define all other variables for which data were sought (e.g. participant and intervention characteristics, funding sources). Describe any assumptions made about any missing or unclear information	Lines
Study risk of bias assessment	11	Specify the methods used to assess the risk of bias in the included studies, including details of the tool(s) used, how many reviewers assessed each study and whether they worked independently, and if applicable, details of automation tools used in the process	Lines 165-169
Effect measures	12	Specify for each outcome the effect measure(s) (e.g. risk ratio, mean difference) used in the synthesis or presentation of results	Lines 151-160
Synthesis methods	13a	Describe the processes used to decide which studies were eligible for each synthesis [(e.g. tabulating the study intervention characteristics and comparing against the planned groups for each synthesis (item #5)]	Lines 143-160
	13b	Describe any methods required to prepare the data for presentation or synthesis, such as handling of missing summary statistics, or data conversions	Lines 143-160
	13c	Describe any methods used to tabulate or visually display the results of individual studies and syntheses	Lines 143-160
	13d	Describe any methods used to synthesize results and provide a rationale for the choice(s). If meta-analysis was performed, describe the model(s), method(s) to identify the presence and extent of statistical heterogeneity, and software package(s) used	Lines 143-160
	13e	Describe any methods used to explore possible causes of heterogeneity among study results (e.g. subgroup analysis, meta-regression)	Lines 143-160
	13f	Describe any sensitivity analyses conducted to assess the robustness of the synthesized results	Lines 143-160
Reporting bias assessment	14	Describe any methods used to assess the risk of bias due to missing results in a synthesis (arising from reporting biases)	Lines 143-160
Certainty assessment	15	Describe any methods used to assess certainty (or confidence) in the body of evidence for an outcome	Lines 143-160
Results			
Study selection	16a	Describe the results of the search and selection process, from the number of records identified in the search to the number of studies included in the review, ideally using a flow diagram	Lines 170-176
	16b	Cite studies that might appear to meet the inclusion criteria, but which were excluded, and explain why they were excluded	Lines 170-176
Study characteristics	17	Cite each included study and present its characteristics	Lines 179-182
Risk of bias in studies	18	Present assessments of risk of bias for each included study	Lines 230-241
Results of individual studies	19	For all outcomes, present, for each study: (a) summary statistics for each group (where appropriate) and (b) an effect estimate and its precision (e.g. confidence/credible interval), ideally using structured tables or plots	Lines 249-266
Results of syntheses	20a	For each synthesis, briefly summarise the characteristics and risk of bias among contributing studies	Lines 249-266
	20b	Present results of all statistical syntheses conducted. If meta-analysis was done, present for each the summary estimate and its precision (e.g. confidence/credible interval) and measures of statistical heterogeneity. If comparing groups, describe the direction of the effect	Lines 249-266
	20c	Present results of all investigations of possible causes of heterogeneity among study results	Lines 249-266
	20d	Present results of all sensitivity analyses conducted to assess the robustness of the synthesized results	Lines 249-266
Reporting biases	21	Present assessments of risk of bias due to missing results (arising from reporting biases) for each synthesis assessed	Lines 249-266
Certainty of evidence	22	Present assessments of certainty (or confidence) in the body of evidence for each outcome assessed	Lines 249-266
Discussion			
Discussion	23a	Provide a general interpretation of the results in the context of other evidence	Lines 270-340
	23b	Discuss any limitations of the evidence included in the review	Lines 341-350
	23c	Discuss any limitations of the review processes used	Lines 341-350
	23d	Discuss the implications of the results for practice, policy, and future research	Lines 351-375
Other information			
Registration and protocol	24a	Provide registration information for the review, including the register name and registration number, or state that the review was not registered	Lines 99-102
	24b	Indicate where the review protocol can be accessed, or state that a protocol was not prepared	Line 102
	24c	Describe and explain any amendments to information provided at registration or in the protocol	Line 100-102
Support	25	Describe sources of financial or non-financial support for the review, and the role of the funders or sponsors in the review	None
Competing interests	26	Declare any competing interests of review authors	None
Availability of data, code, and other materials	27	Report which of the following are publicly available and where they can be found: template data collection forms; data extracted from included studies; data used for all analyses; analytic code; any other materials used in the review	Not applicable
